# Assessment of the Acceptable Range of Lips and Chin Position in Two Different Geographical Zones of Iran among Laypersons

**DOI:** 10.30476/dentjods.2023.97251.2003

**Published:** 2024-06-01

**Authors:** Farzaneh Golfeshan, Athar Nasseri Mojarad, Ahmad Reza Sardarian

**Affiliations:** 1 Dept. of Orthodontics, School of Dentistry Orthodontic Research Center, Shiraz University of Medical Sciences, Shiraz, Iran; 2 Orthodontics Research Center, School Dentistry, Mashhad University of Medical Sciences, Mashhad, Iran

**Keywords:** Lip position, Chin position, Soft tissue profile

## Abstract

**Statement of the Problem:**

The position of the chin and lips are important components in the lower third of the face and can be changed by orthodontic treatment. It seems that factors such as diversity in culture, ethnicity, and place of residence are among the factors affecting people's perception of beauty. Iran, as a vast country, contains different ethnicities and cultures, and of course, it is not exempt from this point of view.

**Purpose:**

Our purpose of conducting this study is to investigate the impact of the difference in living environment and culture on people's aesthetic perception. Orthodontists and oral surgeons can use these data to choose the best treatment plan for the patients according to their geographical zones.

**Material and Method:**

A descriptive cross-sectional study was conducted to evaluate the perception of lips and chin position. A series of 25 profile images were prepared in 5 sets. Each set contained 5 profile images. Northern and Southern lay people and orthodontists were asked to evaluate the profile series of each set in 1 session and score them from 1 to 5: 1, very unattractive; 2, unattractive; 3, neither attractive nor unattractive; 4, attractive; or 5, very attractive.

**Results:**

652 participants in 3 groups, including 16 orthodontists (10 men and 6 women), 318 lay people of the North of Iran (172 men and 138 women), and 318 lay people of the South of Iran (175 men and 139 women) participated in this study. Regardless of the chin position, normal lip position and slight changes of that (in both protruded and retruded positions) were more favorable for all three groups. The images with moderately retruded lips were scored as the least attractive by all three groups and orthodontist gave the lowest score to these profiles. Southern people could better tolerate moderately retruded lips than other two groups. In the fifth series, orthodontists preferred slightly and moderately protruded lips in comparison to other two groups of laypeople.

**Conclusion:**

Regardless of the chin position, normal and slightly (-2mm to +2mm) protruded and retruded lips were more favorable in all three groups. Southern people could better tolerate moderately retruded lips than the two other groups.

## Introduction

The esthetic aspect of the face has become a primary area of focus in our society as people search for ways to improve their facial beauty in the present and over the long-term. Due in part to this relatively recent surge in beauty and esthetics, orthodontists have begun to pay particular attention to the facial profile and soft tissues when evaluating a patient for treatment [ 1 - 2 ]. 

Facial beauty and achieving a balanced lip position in relation to the nose as well as chin and an efficient occlusion have long been known as the goals of orthodontic treatment. The shape of the soft tissue of the face largely determines the aesthetics of the face and is a final compensatory factor in the contour and morphology of the face. For this reason, analysis of these tissues is essential for proper diagnosis and planning for an effective treatment. The range of facial soft tissue is determined by a variety of factors, including the underlying hard tissue, the dental support system, and soft tissue components including the nose, lips, and chin [ 1 - 2 ]. 

In order to create the concept of balance or proportion, hypothetical lines are drawn from several landmarks and different parts of the face are measured relative to each other to determine the amount of imbalance. The three main components, including the nose, lips, and chin, are evaluated in profile view [ 2 ]. 

The position of the lips is one of the most important components in the lower third of the face and can be changed by orthodontics. Lips are an important component in facial beauty and can affect the acceptance and perception of the beauty of the nose and chin. Protruded or retruded lips can affect the patient's main treatment plan and stabilize and function after treatment [ 2 - 3 ]. 

Another part of the face that is especially important in the beauty of the face is the chin. Aesthetically, the chin should have a firm structure, not too prominent or retruded the dimension of the chin should be proportional to other components of the face and this is very important [ 3 ]. The beauty of the chin, nose, and lips are closely related to each other, especially in the profile of the face. If the chin does not grow normally, the mouth seems protruded and looks forward, and lip movements are not normal. A small chin also makes the nose or throat look prominent [ 2 - 3 ]. 

The condition of the lips and chin is affected by the position of the anterior teeth, the skeletal pattern, the size of the nose, and the thickness of the soft tissue. Correction of any of these factors will change the position of the lips and chin and eliminate or reduce facial deformity. The ways to achieve facial beauty and correct abnormalities include growth modification treatments, dental camouflage, and orthognathic surgery [ 1 - 3 ]. 

The Perception of beauty is highly depended on variety of factors such as cultural influences, sex, geographical zone where a man lives, Inheritance, maturity and so on. In order to have an appropriate treatment plan, the patient preference should be considered carefully from the first steps of decision-making [ 1 , 3 ]. 

According to Sena *et al*. [ 4 ] the antero posterior position of the chin exerted strong influence on facial attractiveness, but few significant differences were observed among the different groups of evaluators. 

Alam *et al*. [ 5 ] have concluded that an average profile of the jaw and lips are desired more than retrusive or protrusive profiles among Bangladeshi laypersons. 

In another study, Modarai *et al*. [ 6 ] stated that the most important factor in profile attractiveness was the amount of sagittal discrepancy, chin protrusion was less attractive than retrusion, and surgery was desired more often for these images. 

Zarif najafi *et al*. [ 7 ] evaluated the preference of lip projection among 5 panel people including 79 senior dentistry students, 26 orthodontists, 27 maxillofacial surgeons, 27 prosthodontists, and 81 laypeople. The results of his study showed that a significant difference was presented between raters in ranking of number of images while the gender difference was not considered to be an influencing factor in answering the questionnaires.

It seems that factors such as diversity in culture, ethnicity, and place of residence are among the factors affecting people's perception of beauty [ 8 ]. Iran, as a vast country, contains different ethnicities and cultures, and of course, it is not exempt from this point of view.

Our purpose of conducting this study was to investigate the impact of the difference in living environment and culture on people's aesthetic perception. The objective of this study was to compare the lips and chin position preference among geographically apart lay people. A clinician must fully understand the patient's wishes in order to manage expectations and achieve a result close to what the patient desires. Hence the purpose of the present study was to evaluate the preferred lip and chin position, determine if geographical zones contribute to lip and chin position preferences, develop soft tissue “norms” or standards which may be a useful guide for diagnosis and treatment planning in orthodontic patients and to finally Assess and compare the esthetic lip preferences of orthodontists and the lay public in straight, retrognathic and prognathic profile. 

## Materials and Method

This descriptive study was approved in ethics committee of Shiraz University of Medical Science with the ethical code IRSUMS DENTAL.REC1399.072. The sample size of this study was 550 people based on a similar study conducted by Taee A *et al*. [ 9 ] in 2019 assuming alpha .05 and beta 80% and 10% possible dropout. A standard silhouetted image was constructed using Photoshop software (Photoshop CS; version 8.0 Adobe systems, San Jose, Calif) with reference to normal soft tissue values as presented in Table1. 

**Table 1:** Normal soft tissue profile characteristics

1. Equal facial thirds in vertical dimension (1: from the region which hair growth to the most prominent point of the forehead. 2: from the most prominent portion of the forehead to the subnasion. 3: from the subnasion to the most prominent point of the chin.)
2. In the inferior facial third, the upper lip vertical dimension must be half of the lower lip and chin vertical dimension together.
3. Lips relation with E line: upper lip must be 3mm and the lower lip 2mm behind the E line.
4. Lips relation to the S line: the upper lip must be tangent to the line and the lower lip must be 1mm behind it.
5. Facial angle: the angle between the Frankfort line and the N-Pog connecting line is considered as 90 degrees.
6. Nasal forehead angle: the angle between the N-G connecting line and the N-Pn connecting line is considered as 126 degrees.
7. nose-lip angle: the angle between the line tangent to the inferior border of nose and the most prominent part of the upper lip must be 92 degrees.
8. facial-nose angle; the angle between the N-Pn and N-POG connecting lines must be 34 degrees.
9. mental-nose angle: the angle between the two N-Pn and Pn-Pog connecting line must be 128 degrees.
10. mento-labial angle: the angle between the lower lip and the chin must be 120 degrees.
11. chin-neck angle: the angle between the G-Pog connecting line and the line tangent to the inferior border of the chin must be 90 degrees.
12. throat-neck angle: the angle between the throat tangent line and the neck tangent line must be 110 degrees.

To evaluate the perception of lips and chin position a series of 25 profile images were prepared in 5 sets. Each set comprised 5 profile images. 

### Set 1

**Figure 1:**
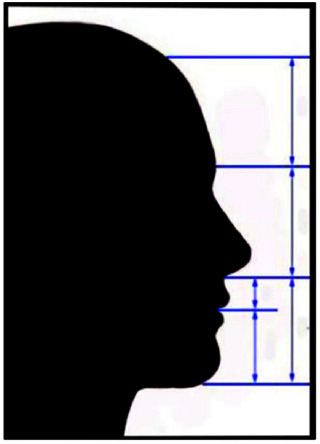
Equal vertical one thirds of the face and the proportions of the lower one third

**Figure 2:**
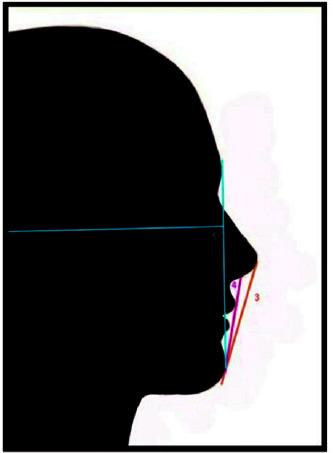
Lips to the E line (3) lips to the S line (4)

**Figure 3:**
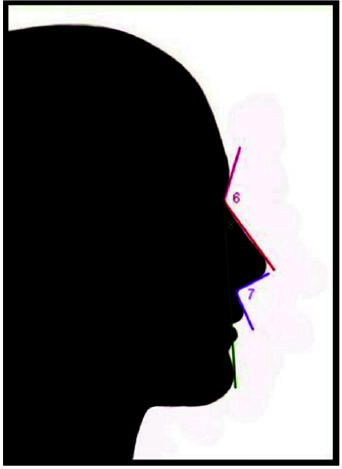
Nasal forehead angle (6) nose lip angle (7) facial nose

**Figure 4:**
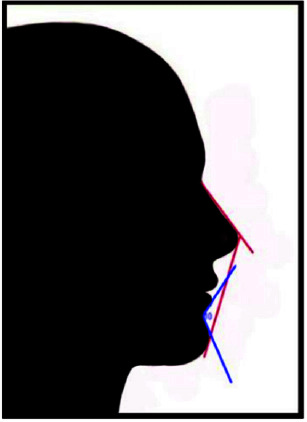
Mental nose angle (9) mentolabial angle (10)

**Figure 5:**
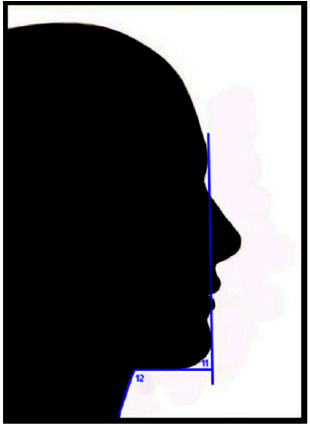
Chin neck angle (11) throat neck angle (12)

**Figure 6:**
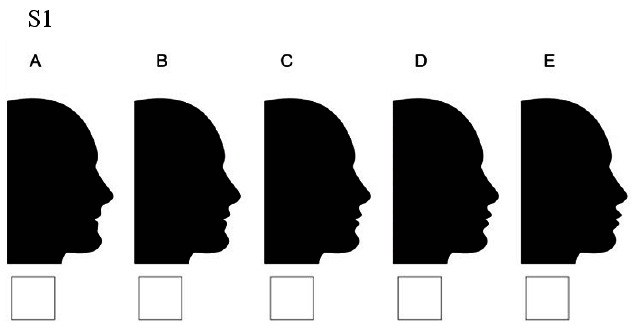
Lips displacement while the chin is in normal position (S1)

S1A: lips moved 4 millimeters backward/S1B: lips moved 2 millimeters backward/S1C: lips and chin at the normal position/ S1D: lips moved 2millimeters forward/ S1E: lips moved 4 millimeters forward (Figures 1-6). 

### Set 2

**Figure 7:**
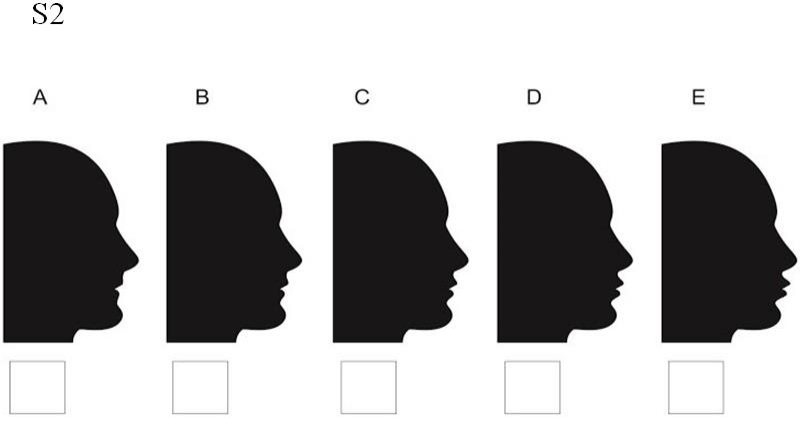
Lips displacement while the chin is 2 millimeters retruded (S2)

S2A: lips moved 4millimeters backward and chin moved 2 millimeters backward/ S2B: lips moved 2 millimeters backward and chin moved 2 millimeters backward/ S2C: chin moved 2 millimeters backward but lips stay at the normal position/ S2D: chin moved 2 millimeters backward and lips moved 2 millimeters forward/S2E: chin moved 2 millimeter s backward and lips moved 4 millimeters forward (Figure 7). 

### Set 3

**Figure 8:**
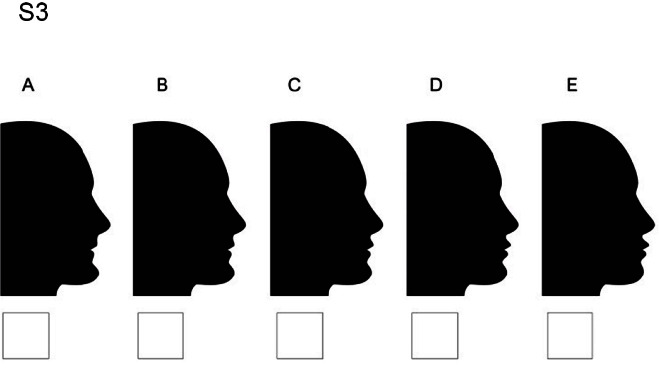
Lips displacement while the chin is 2 millimeters protruded (S3)

S3A: lips moved 4 millimeters backward and chin moved 2 millimeters forward/ S3B: lips moved 2 millimeters backward and chin moved 2 millimeters forward/ S3C: chin moved 2 millimeters forward but lips stay at the normal position/ S3D: chin moved 2 millimeters forward and lips moved 2 millimeters forward/S3E: chin moved 2 millimeters forward and lips moved 4 millimeters forward (Figure 8). 

### Set 4

**Figure 9:**
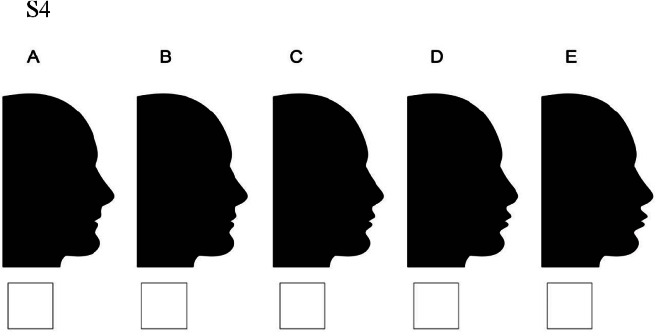
Lips displacement while chin is 4 millimeters retruded (S4)

S4A: chin and lips both moved 4 millimeters backward / S4B: lips moved 2 millimeters backward and chin moved 4 millimeters backward/ S4C: lips stay at the normal position and chin moved 4 millimeters backward/ S4D: lips moved 2 millimeters forward and chin moved 4 millimeters backward/ S4E: lips moved 4 millimeters forward and chin moved 4 millimeters backward (Figure 9). 

### Set 5

S5A: lips moved 4 millimeters backward and chin moved 4 millimeters forward / S5B: lips moved 2 millimeters backward and chin moved 4 millimeters forward/ S5C: lips stay at the normal position and chin moved 4 millimeters forward/ S5D: lips moved 2 millimeters forward and chin moved 4 millimeters forward/ S5E: lips moved 4 millimeters forward and chin moved 4 millimeters forward. 

The Likert index is one of the efficient and reliable indicators in the field of psychology . 

**Figure 10:**
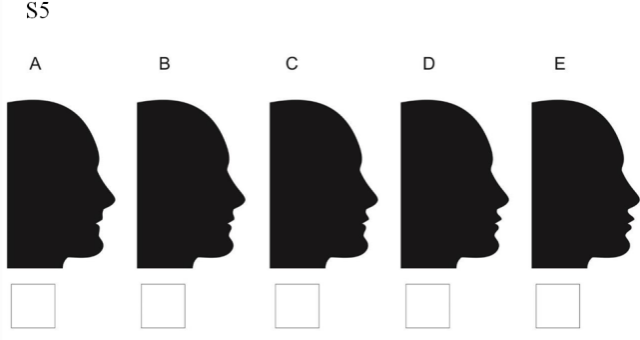
Lips displacement while chin is 4 millimeters protruded

Raters (laypersons) were randomly chosen from people whom were referred to governmental clinics, which were selected randomly between all centers (Figure 10). 

Adults with at least 18 years of age were invited to participate in the study. Those who had previously received orthodontic treatment or had undergone orthognat-hic surgery, patients with a history of head and neck pathology or trauma and also those who were engaged in health care occupations, were excluded from the study. 

All raters are homogenized for the above attributes and the only exception, which differs between the two groups, is the geographical region which one lives in.

The orthodontist raters were our colleagues and their collaborators whom were up to work at the two segregated geographically areas. All the respondents were asked to carefully observe and rate the pictures in each of the groups from 1 to 5: 1, very unattractive; 2, unattractive; 3, neither attractive nor unattractive; 4, attractive; or 5, very attractive. The questionnaire also included questions related to the respondents' demographic information such as age, sex, and their profession. During the process of answering the questionnaires, each person was asked to answer the questions within ten minutes in a quiet place with high concentration. Then each completed questionnaire was named with numerical codes in order to be anonymized. Finally, twenty percent of the respondents were asked to answer the questionnaires again after two weeks in order to be sure of the reliability of the answers. 

### Statistical analysis

Statistical analysis was performed using SPSS software (version 19; IBM, Armonk, NY). The means and standard deviations of all images rank scores were calculated. In addition, the means and standard deviations of each group rank scores were calculated independently. In order to compare the rankings of the images among all groups, the Kruskal-Wallis test was used. For comparing the scores of the whole raters and pair-wise comparisons in all groups, the Mann-Whitney test was used. Intraclass correlation coefficients (ICCs) with a 95% confidence interval were tested to check the reproducibility among scores between the two evaluations. 

## Results

In this study, out of 670 questionnaires distributed, 652 questionnaires were completed and 18 people did not complete the questionnaire and participate in the study (97% response rate). A total of 652 participants in 3 groups, including 16 orthodontists (10 men and 6 women), 318 lay people of the North of Iran (172 men and 138 women) and 318 lay people of the South of Iran (175 men and 139 women) participated in this study. Table 2 shows the means and standard deviations of the scores given to the images by all three groups. This test was done separately for our Northern and Southern groups of orthodontists and none of the findings were statistically significant therefore, we considered the two groups as one group named orthodontist group. 

**Table 2:** Mean and standard deviation of the scores given to set of images by participants and Pair-wise comparisons of the profile images that received significantly different mean scores by different groups

Set of Images	North (A)	South (B)	Orthodontists (C)	A-B	A-C	B-C
S1a	1.79±1.2	2.49±1.6	1±0	SIG	SIG	SIG
S1b	3.05±1.1	3.04±1.2	2.13±.3	------	SIG	SIG
S1c	3.75±1.2	3.40±1.3	4.63±.5	SIG	SIG	SIG
S1d	3.47±1	3.18±1.3	4.13±.7	SIG	SIG	SIG
S1e	2.86±1.4	2.88 ±1.3	3.13±.7	------	-----	------
S2a	1.98±1.2	2.57 ±1.4	1±0	SIG	SIG	SIG
S2b	3.27±1.2	3.03 ±1.2	3.38±1	SIG	-----	------
S2c	3.80±1.2	3.44±1.4	4.50±.6	SIG	SIG	SIG
S2d	3.46±1.1	3.29±1.2	3.94±.7	------	-----	SIG
S2e	2.33±1.3	2.64± 1.4	2.19±.4	SIG	-----	------
S3a	1.74±1.2	2.33 ±1.4	1.13±.3	SIG	-----	SIG
S3b	2.89±1.1	2.83±1.2	2.63±.6	------	-----	------
S3c	3.90±1.2	3.42 ±1.3	4.63±.5	SIG	SIG	SIG
S3d	3.47±1.1	3.36±1.3	4.31±.6	------	SIG	SIG
S3e	2.86±1.3	3.05±1.3	2.44±.6	-------	-----	------
S4a	2.22±1.3	2.60±1.4	1.56±.5	SIG	-----	SIG
S4b	3.37±1.3	3.23±1.3	3±0	-------	-----	------
S4c	3.77±1.1	3.46±1.3	4.63±.5	SIG	SIG	SIG
S4d	3.26±1.1	3.09±1.2	4.44±.5	-------	SIG	SIG
S4e	2.27±1.3	2.61±1.4	1.44±.5	SIG	SIG	SIG
S5a	1.88±1.2	2.36±1.5	1.19±.4	SIG	-----	SIG
S5b	3.20±1.1	3.15±1.2	2.06±.5	-------	SIG	SIG
S5c	3.65±1.2	3.30±1.2	2.93±.5	SIG	SIG	------
S5d	3.43±1.2	3.34±1.3	4.56±.5	------	SIG	SIG
S5e	2.71±1.4	2.82±1.4	4.31±.7	------	SIG	SIG

SIG= Significant

S= series 1-5

a-e= shows the image in each series of silhouette

### Set 1. Normal chin position

In normal chin position (set 1), normal and slightly protruded (+2mm) or retruded (-2mm) lips were the most acceptable ones in all three groups. It should be mentioned that moderately retruded lips (-4mm) were better to-lerated by Southern lay people than Northern ones. Moderately retruded lips (-4mm) and protruded lips (+4mm) was scored as the most unattractive one by orthodontist. 

### Set 2. Slightly retruded (-2mm) chin position

In slightly retruded (-2mm) chin position (Set 2), normal and slightly protruded (+2mm) and retruded (-2mm) lips were the most acceptable ones in all three groups. Moderately retruded lips (-4mm) was the most unattractive one in all three groups. Also similar to the previous set Southern laypeople could better tolerate moderately retruded (-4mm) lips than Northern ones. 

### Set 3. Slightly protruded (+2mm) chin position

In slightly protruded (+2mm) chin position (Set 3) normal and slightly protruded (+2mm) and retruded (-2mm) lips were the most acceptable ones in all three groups. The moderately retruded (-4mm) lips position were the least acceptable one in all three groups. It should be mentioned that Southern laypeople could better tolerate moderately retruded lips than other groups. 

### Set 4. Moderately retruded (-4mm) chin position

In moderately retruded (-4mm) chin position (Set 4) normal and slightly protruded (+2mm) and retruded (-2mm) lips were the most acceptable ones in all three groups. However, orthodontist’s preferences in these profiles were bolder. The least attractive image in view of North and South people was the moderately retruded (-4mm) lips position, while the orthodontist scored the moderately protruded (+4mm) lips as the most unattractive image. In addition, Southern laypeople could better tolerate moderately retruded lips than other groups. 

### Set 5. Moderately protruded (+4mm) chin position

In moderately protruded (+4mm) chin position (Set 5) normal and slightly protruded (+2mm) and retruded (-2mm) lips were the most acceptable ones in both groups of South and North people. While in orthodontists view moderately and slightly protruded (+2, +4mm) lips are the most attractive profiles. Moderately retruded lips (-4mm) was the most unattractive one in all three groups. In addition, Southern laypeople could better tolerate moderately retruded lips than other groups. 

Pair-wise comparisons of the profile images that received significantly different mean scores by different groups were presented in Table 2. 

Since twenty percent of the respondents had completed the questionnaires again, the reliability of the answers was measured by the ICC. The ICC was 0.72 (lower limit, 0.64; upper limit, 0.79, with 95% confidence), which shows a high degree of agreement in completing the questionnaires among the judges when scoring each photograph. 

## Discussion

The goal of orthodontic treatment is to achieve a balanced face through proper soft tissue alignment, tooth stabilization, and occlusion coordination. The position of the lips and chin are factors affecting the balance and beauty of the face, especially in profile [ 3 ]. Dentists face different positions of the lips and chin when diagnosing and treating. These variations can be attributed to a skeletal or anatomical abnormality of the soft tissue [ 3 ]. Orthodontic and surgical treatments can achieve cosmetic goals and improve facial abnormalities by improving maxillofacial relationships, creating an efficient occlusion, designing a beautiful smile arch, and repositioning the soft tissues of the lips and chin [ 11 - 12 ]. 

Our purpose of conducting this study is to investigate the impact of the difference in living environment and culture on people's aesthetic perception. Orthodontists and oral surgeons can use these data to choose the best treatment plan for the patients according to their geographical zones. 

By collecting 652 questionnaires answered by 318 Northern laypeople, 318 Southern laypeople, 16 orthodontists (consist of 8 Northern specialists and 8 Southern ones) containing a series of pictures with different positions of lips and chin in facial silhouettes, we had identified the most desirable profile. 

To limit the influence of components such as hair, skin complexion, and eyes on the facial attractiveness, previous authors had used androgynous silhouettes in order to evaluate the profile esthetics [ 13 ]. It has been stated that factors such as hairstyle instead of the profile outline shape can bias the beauty scores [ 14 ]. Moreover, another study reported that average size of facial features such as large eyes, cheekbones, and chins for men can look more attractive [ 15 ]. However, these factors can interfere in the accurate response of the participants assessing the profiles [ 13 ].

In many cultures, beauty standards have evolved and they are influenced by various factors such as historical events, environments, and even globalization. While some beauty ideals might seem universal due to media influence, others are deeply rooted in local and traditional values [ 8 , 16 - 17 ].

Every culture and ethnicity could influence on how a person is perceived. Various studies had tried to evaluate if facial attractiveness differ between culturally apart groups [ 18 - 19 ]. They had showed that, irrespective to the ethnical backgrounds, respondents preferred the same faces and accepted that certain facial attributes are important in aesthetical perceptions [ 8 ]. This study tried to find out if these findings could be extended to our own society. 

Our study primarily focused on a specific group, the orthodontists. While the results might offer insights about their perceptions of beauty and aesthetics, generalizeng these findings to a broader population may not be accurate. The limitation in sample size, due to accessibility challenges, means that we were unable to compare the perceptions of orthodontists from different geographical regions or compare their views to those of laypeople.

In our opinion, the insignificant findings within the orthodontists group revealed that academic education could defuse the effect of culture or ethnics and we can have considered the two groups of orthodontists as one group irrespective to their different geographical zones. 

In different positions of chin, normal and slightly protruded and retruded lips are more favorable for all three groups. The images with moderately retruded lips were scored as the least attractive by all three groups and orthodontist gave the lowest score to these profiles. Southern people could better tolerate moderately retruded lips than other two groups. In the fifth series, which the chin is moderately protruded, there is a small different in orthodontist’s view with other two groups which showed that, slightly and moderately protruded lips were the most acceptable profiles. 

The perception of beauty is highly depended on variety of factors such as cultural influences, sex, geographical zone, inheritance, maturity, and so on [ 6 , 20 - 21 ].

Many studies [ 6 , 20 - 21 ] had been done previously to assess different factors affecting perception of beauty. Some of these studies tried to compare the concept of beauty between different races [ 18 , 22 ]. In addition, some of these studies [ 15 , 19 , 23 ] mentioned the differences in perception of beauty in different groups of people with different academic degrees or professional occupations. 

In studies conducted by Kamble *et al*. [ 21 ] and Modarai *et al*. [ 6 ], no differences were reported between ordinary people and orthodontists; they had similar views regarding the perception of the aesthetics of the anterior-posterior position of the lips and chin in profile view and no statistically significant difference was observed between them. Naini *et al*. [ 3 ] compared the effect of chin protrusion on aesthetic perception in three groups of orthodontic patients, dentists, and the general p-opulation. In general, the aesthetic views of all three groups were almost similar. In Shimogaki’s study [ 24 ], there was no significant difference between orthodontists and ordinary people in terms of prioritization for both groups. To our knowledge, none of the past studies has assessed the impact of culture and ethnics in perception of beauty. In order to have an appropriate treatment plan, the patient preference should be considered carefully from the first steps of decision making. 

In our study, the most acceptable profile in each set of images was the same for all three groups. These results are encouraging because they show that all groups have common aesthetic concepts, thus increasing the likelihood of patient satisfaction at the end of treatment. However, Foster [ 19 ] reported in his study that orthodontists differ from dentists and the general public in assessing the position of the lips and chin. The findings of Hier *et al*. [ 23 ] also showed that ordinary people have different opinions compared to orthodontists. These differences can be due to education, educational background, or knowledge about facial disorders. 

In our study, the comparison between a group of orthodontists and two other groups, profiles with normal and slightly protruded lips were the most attractive, while ordinary people in the North and South, in addition to profiles with normal and slightly protruded lips, preferred those that had slightly retruded lips. These differences showed that lay people were not sensitive for lips position in the range of -2 to +2mm, while it makes differences for orthodontists. 

In the comparison between the two regions of the North and the South, we came to the conclusion that ordinary people in the South of the country could tolerate moderately retruded lips to a greater extent. 

As we know in surgical and orthodontic treatments, therapists try to make a harmonic condition in anteroposterior profile between lips, chin, and the nose. 

The size and the shape of the nose and also the chin are factors which could be modified by surgeries. In extremely retruded or protruded chin, treatments such as advancement genioplasty and reduction genioplasty are the only efficient ways in adult patients [ 25 - 26 ]. Lip position can be changed by orthodontic treatment or cosmetic surgery like lip augmentation. Orthodontic treatments could have control on lips position by changing the inclination of incisor teeth by extraction or non-extraction treatments [ 27 ]. Orthodontic treatments that involve tooth extraction can lead to changes in facial profile. Specifically, we observed a decrease in a particular angle, leading to a more retruded appearance of the lips.

In some cases, the chin position is slightly far from the ideal norms in either retruded or even protruded direction. In this situation, surgical treatments are not actually the best choice and most of the patients do not totally accept them. This is the condition in which orthodontic treatments could help the therapist make harmonic and balanced profile by changing the lips positions based on the degree of chin prominence. For example, in retruded chins, lips with less prominence could be better tolerated [ 3 , 21 ]. 

Considering the differences between the findings of the North and the South, it can be concluded that in the South of the country, patients are less sensitive to retruded lips. In this region, extraction treatment that causes reduction in lip prominence can be better tolerated with different chin position. 

In our study, there were no intention for assessing the differences in facial beauty preference within sexes while number of studies had gone over that and some not [ 6 ]. 

Modarai *et al*. [ 6 ], examined the effect of lower lip position on the perception of the attractiveness of chin protrusion, however, they did not report a significant difference between the aesthetic views of men and women. In addition, in the research of Zarif Najafi *et al*. [ 7 ] on the effect of lip position on the beauty of facial profile, no significant difference was seen between the mean scores of the two genders. It is important to note the absence of gender bias in determining the most appealing anterior-posterior position of the lips and chin. This could indicate that many societies draw their standards of facial beauty from shared influences, irrespective of gender. Factors like culture, economy, societal norms, and possibly even more unidentified elements play a role in shaping these shared standards [ 28 ]. 

However, Hier *et al*. [ 23 ], after examining priorities and opinions about different lip positions, stated that women prefer more prominent lips than men do. The difference between their results and the results of the current study might be due to the different methods used to depict profile views. 

We had some limitations during our studies, which were, corona virus outbreak following that long quarantines and data gathering difficulties and also the complexities of statistical analysis, especially in the category of gender analysis and its impact on aesthetic preferences. 

In future studies, a larger number of orthodontic specialists and even colleagues from other related specialties such as maxillofacial surgeons are suggested to be surveyed so that a wider comparison could be made.

## Conclusion

In different positions of chin, normal and slightly protruded and retruded lips are more favorable for all three groups. The images with moderately retruded lips were scored as the least attractive by all three groups and orthodontist gave the lowest score to these profiles. Southern people could better tolerate moderately retruded lips than other two groups. In the fifth series, in which the chin was moderately protruded there was a small different in orthodontist’s view with other two groups which showed that slightly and moderately protruded lips are the most acceptable profiles. 
